# *k*-core genes underpin structural features of breast cancer

**DOI:** 10.1038/s41598-021-95313-y

**Published:** 2021-08-11

**Authors:** Rodrigo Dorantes-Gilardi, Diana García-Cortés, Enrique Hernández-Lemus, Jesús Espinal-Enríquez

**Affiliations:** 1 Network Science Institute and Department of Physics, Northeastern University, Boston, MA 02115 USA; 2 El Colegio de México, Tlalpan, Mexico City, 14110 Mexico; 3 Computational Genomics Division, National Institute of Genomic Medicine (INMEGEN), Mexico City, 14610 Mexico; 4 Centro de Ciencias de la Complejidad, Universidad Nacional Autónoma de México (UNAM), Mexico City, 04510 Mexico

**Keywords:** Breast cancer, Gene regulatory networks, Cancer genomics

## Abstract

Gene co-expression networks (GCNs) have been developed as relevant analytical tools for the study of the gene expression patterns behind complex phenotypes. Determining the association between structure and function in GCNs is a current challenge in biomedical research. Several structural differences between GCNs of breast cancer and healthy phenotypes have been reported. In a previous study, using co-expression multilayer networks, we have shown that there are abrupt differences in the connectivity patterns of the GCN of basal-like breast cancer between top co-expressed gene-pairs and the remaining gene-pairs. Here, we compared the top-100,000 interactions networks for the four breast cancer phenotypes (Luminal-A, Luminal-B, Her2+ and Basal), in terms of structural properties. For this purpose, we used the graph-theoretical *k*-core of a network (maximal sub-network with nodes of degree at least *k*). We developed a comprehensive analysis of the network k-core ($k=30$) structures in cancer, and its relationship with biological functions. We found that in the Top-100,000-edges networks, the majority of interactions in breast cancer networks are intra-chromosome, meanwhile inter-chromosome interactions serve as connecting bridges between clusters. Moreover, core genes in the healthy network are strongly associated with processes such as metabolism and cell cycle. In breast cancer, only the core of Luminal A is related to those processes, and genes in its core are over-expressed. The intersection of the core nodes in all subtypes of cancer is composed only by genes in the chr8q24.3 region. This region has been observed to be highly amplified in several cancers before, and its appearance in the intersection of the four breast cancer *k*-cores, may suggest that local co-expression is a conserved phenomenon in cancer. Considering the many intricacies associated with these phenomena and the vast amount of research in epigenomic regulation which is currently undergoing, there is a need for further research on the epigenomic effects on the structure and function of gene co-expression networks in cancer.

## Introduction

As is widely recognized structure determines, to an extent, function in biological networks^1–3^. It is also known that in cancer, gene regulation is severely affected by mutation, changes in gene expression, signaling, and metabolic processes^4–8^. In gene co-expression networks, the strongest gene–gene interactions may extert a large influence in the regulatory landscape^9–12^. However, it is not easy to determine the quantitative threshold to consider an interaction as valid. An appealing manner of detecting the set of genes that may determine the structure of the network is by observing the *k-core*, i.e., the set of nodes that have at least *k* neighbors. The manner in which k-core genes are connected may underlie the structure and function of a large part of the network^13–15^.

We have observed a particular phenomenon in breast cancer gene co-expression networks: inter-chromosome interactions have low values in breast cancer, and the largest co-expression values—as computed by the mutual information (MI) statistical dependency measure on their expression profiles—occur between physically close genes (same chromosome, same cytoband)^16–19^. For the healthy network case, gene–gene interaction strength (highest MI) is almost independent of the chromosome or position of genes. This phenomenon has been also observed very recently in clear cell renal carcinoma^20^, lung aednocarcinoma and squamous cells lung carcinoma^21^. It is worth mentioning that the loss of long-distance co-expression strength occurred in the top (higher MI) gene co-expression interactions.

In order to have a more detailed description of the phenomenon, a previous work by our group^14^, discuss the difference between basal breast cancer and healthy networks in the whole range of interactions. By splitting the complete set of transcriptional relationships ($\simeq$ 200 million) into consecutive sets of 100,000 interactions, we observed that in the healthy network, there is a set of genes that determines the structure of networks throughout several layers, being the same set in all of them. In the case of the cancer network, the set changes almost completely in the first layers of interactions^14^.

Additionally, the identity of genes in the *k-core* in the healthy network remains along dozens of layers, however in the case of cancer networks, the *k-core* changes from the second layer. This fact indicates that the first set of interactions in cancer networks is not fully *permeating* the subsequent layers. In other words, the first layer mostly determines the regulatory landscape in cancer.

Based on these premises, in an attempt to have a comprehensive analysis of the core structure of networks, the chromosomal location and their functional implications, we analyzed the *k-core* networks with a $k=30$ for all phenotypes in the first layer. We constructed the networks, analyzed their structural parameters, and observed the chromosomes to which those genes belong. We performed community detection, in order to find the most interconnected sets, as well as their connectivity features. Additionally, we performed a differential gene expression analysis to observe whether or not the cancer *k-*cores had a differential expression trend, in terms of connectivity. We also performed a functional enrichment analysis of the detected communities, to find the biological functions in which those interconnected genes may participate together.

## Methods

### Data acquisition

The complete collection of The Cancer Genome Atlas (TCGA)^22^ breast RNA-Seq samples was downloaded in January, 2019 from the GDC repository https://portal.gdc.cancer.gov/repository. This collection included 113 solid tissue normal samples and 1102 primary tumor samples.

### Data pre-processing

#### Data integration

An integrity check was carried out in raw expression files using gene annotations from BioMart. Only protein coding genes belonging to conventional chromosomes (1, 2,..., 22, X and Y) were kept.

#### Quality control

NOISeq R library was used for global quality control in order to assess several aspects^23,24^. First, the relative biotype abundance in the experimental conditions were evaluated in order to assess if samples contained protein coding expression genes in their majority. Second, gene counts expression boxplots were evaluated per biotype to confirm that the highest median expression corresponded to protein coding genes. Third, saturation plots were obtained, i. e., the number of detected genes (counts > 0) per sample across different sequencing depths as simulated by NOISeq.

All samples reached saturation for the number of detected features at the corresponding sequencing depth, i.e., no further gene will be detected. Fourth, global expression quantification for each experimental condition yielded a feature sensitivity > 60% for 10 count per million (CPM), which suggests an accurate library preparation. Fifth, different bias detection plots were tested, where bins containing the same number of corresponding ordered genes based on their mean gene length, %GC and RNA content were plotted against their corresponding mean expression of gene counts.

EDASeq R library was used for batch effect removal^25^. Before normalization genes with mean counts < 10 were filtered, as suggested in Ref.^25^. Different within/between normalization strategies were tested to remove bias presence. The best alternative was sequentially full quantile GC content and gene length within normalization followed by Trimmed Mean of M values (TMM)^26^ between normalization. Within full quantile normalization consisted in matching the distribution of the gene counts to a reference distribution defined in terms of median counts across the artifact to be removed (%GC or gene length) for each sample. Between normalization using TMM assumes that the majority of the genes is not differentially expressed and empirically equates the overall gene expression levels between samples based on a reference sample. The TMM scaling factor value for each sample is a weighted sum of the log-fold change of each gene with respect to the reference sample, with weights as the inverse of its approximate asymptotic variance. However, this sum is trimmed in the sense that it uses only the genes that were not present in the lower and upper 30%/5% of log-fold change and average expression values respectively. Afterwards, NOISeq analysis confirmed artifacts removal.

The code for data pre-processing can be found in the following repository: https://github.com/CSB-IG/regulaciontrans-pipeline.

### Data processing (tumor samples)

The tumor $log_2$ normalized expression values were classified using PAM50 algorithm into the respective intrinsic breast cancer subtypes (Normal-like, Luminal A, Luminal B, Basal and HER2-Enriched) using the Permutation-Based Confidence for Molecular Classification^27^ as implemented in the *pbcmc* R package^28^. Tumor samples with a non-reliable breast cancer subtype call, were removed from the analysis. The number of reliable samples were 113, 217, 192, 105, and 221 for control, Luminal A, Luminal B, HER2+, and Basal subtypes, respectively. Multidimensional Principal Component Analysis (PCA) over gene expression values showed a blurred overlapped pattern among the different breast cancer subtypes. Hence, multidimensional noise reduction using ARSyN R implementation was used as in the control sample^29^. Finally, PCA visual exploration showed that the noisy pattern was removed, thus breast cancer subtypes clustered without overlap.

### Network construction

Gene regulatory network deconvolution from experimental data has been extensively used to unveil co-regulatory interactions between genes by looking out for patterns in their experimentally-measured mRNA expression levels^14,19^. Several correlation measures have been used to infer transcriptional interaction networks^30–34^.

It is largely known that the maximum likelihood estimator of statistical dependency is mutual information (MI)^33–36^. For the construction of the five phenotype networks analyzed in this work, we used ARACNE^37^, an algorithm that quantifies the statistical dependence between pairs of genes. It is based on the calculation of the pairwise mutual information (MI) function for the expression vectors of every gene. Significance analysis of the MI distributions is made via Chow–Liu graph-theoretical methods and permutation analysis^35,37^. This method has been applied broadly to infer and analyze gene regulatory networks in general^38–42^, as well as cancer gene ragulatory networks^43–47^. Despite other computational methods have been developed to calculate mutual information, such as infotheo^48^, entropy^49^, or Scikit-learn^50^, ARACNE allows multi-threading and is a low-consuming algorithm in terms of computational resources.

As in Ref.^14^, we consider 101 equally-sized co-expression intervals containing 0.1% of the co-expression values each. Each layer contains approximately 104,000 interactions. Each layer contains a unique set of gene–gene non-overlapping interactions, however, they may have the same genes. All layers have thus the same number of edges but not necessarily the same number of nodes.

Under this context, we speak interchangeably of interactions, transcriptional relationships, and edges between genes. A sharing of an edge is defined only by a measure of mutual information and corresponds to two genes having a similar pattern of expression across samples. This may increase the probability of the two genes being part of a same transcriptional regulatory program or pathway. The definition of interaction is thus simply related to high mutual information between gene pairs but may have a biological association as argued above.

As stated, statistical dependencies among genes are actually significant. On the technical side, undirected networks based on pairwise statistical dependencies, belong to a class of probabilistic graphical models called Markov random fields (MRFs). As such, MRFs are indeed “learning machines”. In this (information theoretical) sense, co-dependent random variables “communicate” via their dependency structures, since by virtue of these dependencies, a change in one random variable will induce (in the probabilistic sense) a change in any of their network neighbors by virtue of the so-called pairwise (or local) Markov property.

On a related subject, a co-expression layer is defined on a range of mutual-information, and is related to a same level of co-expression of 100,000 gene-pair interactions. The top-most co-expression layer is related to the 100,000 stronger co-expressed gene interactions in the data, and is the main layer of study in this work.

### Differential expression analysis

Differential expression analysis was performed as described in Ref.^16^. In sum, the limma package^51^ in R was used to determine over-expressed or under-expressed genes, by adjusting a gene-based linear model. An absolute difference of log2 fold change $\ge 0.5$ and a *p-value* $< 0.05$ was set as threshold.

### *k*-core genes

The *k-core* is defined as follows: given a network $G=(V, E)$ with set of nodes *V* and edges *E*, a *k*-core is a maximal subgraph of *G* such that every node in the subgraph has degree at least *k*. To observe whether or not *k-core* genes are preserved through the different layers in all phenotypes, we used the cumulative conservation rate *ccr*, previously defined in Ref.^14^. The *ccr* of the top layer ($ccr_{100}$) is always equal to 1, as core genes are taken from layer 100. Let $S_i$ be the set of core genes in the layer *i*, we define $ccr_{99} = |S_{99} \cap S_{100}|/|S_{100}|$, where ‘|*S*|’ denotes the number of nodes in set *S*.

For any two layers *i*, *j*, where $j < i$, we have that $ccr_j \le ccr_i$. The slower the decay of *ccr* from top to bottom layer, the more conserved is the initial group of nodes. The conservation of a set of nodes across co-expression layers, could indicate its relevance in cell regulatory processes at different co-expression scales, and possibly also in the global network structure.

As previously mentioned, the *k-core* of a network is the maximal sub-network with vertices of degree at least *k*. Evidently, the *k-core* in each network may be different. Hence, in order to make all phenotype networks comparable, we set the *k* for all networks in $k=30$. The *30-core* is therefore the maximal sub-network with vertices having at least 30 neighbors.

For this case, we kept the *k-core* of the first layer in order to analyze comprehensively the most important genes in terms of its structtural features, and observe whether or not these genes are conserved along layers, and also whether these genes and its conocomitant community structure have functional implications.

In order to select a core number that yields similar size network, we used the 30-core of each cancer subtype network. For each network, the 30-core is composed of roughly 5% of nodes (Supplementary Fig. S1). Indeed, the mean 0.95 quantile of the core number distribution across the 4 cancer subtypes is 30.25. Supplementary Data contains the interactions obtained by each 30-core network for each one of the five phenotypes.

### Community detection

To have a more detailed description of the core networks structure, we performed the community detection method of Girvan–Newman fast greedy algorithm^52^, implemented in Glay^53^ app of Cytoscape v.8.1.0^54^. Briefly, the algorithm uses the edge betweenness measure to optimize modularity, based on the idea that edges with high betweenness connect modules. Iterative removal of high betweenness edges provides the resultant communities in the network cores.

### Overrepresentation analysis

To identify over-represented or enriched terms in the Biological Process category in Gene Ontology (GO), the gProfiler function^55^ was implemented. Enrichment analysis was performed for all detected communities. We conserved those GO-terms with adjusted adj.* p*-val < 10^−5^, using the Benjamini and Hochberg method for multiple testing.

## Results

### First layer cancer GCNs show a remarkable *cis-*preference

**Figure 1:**
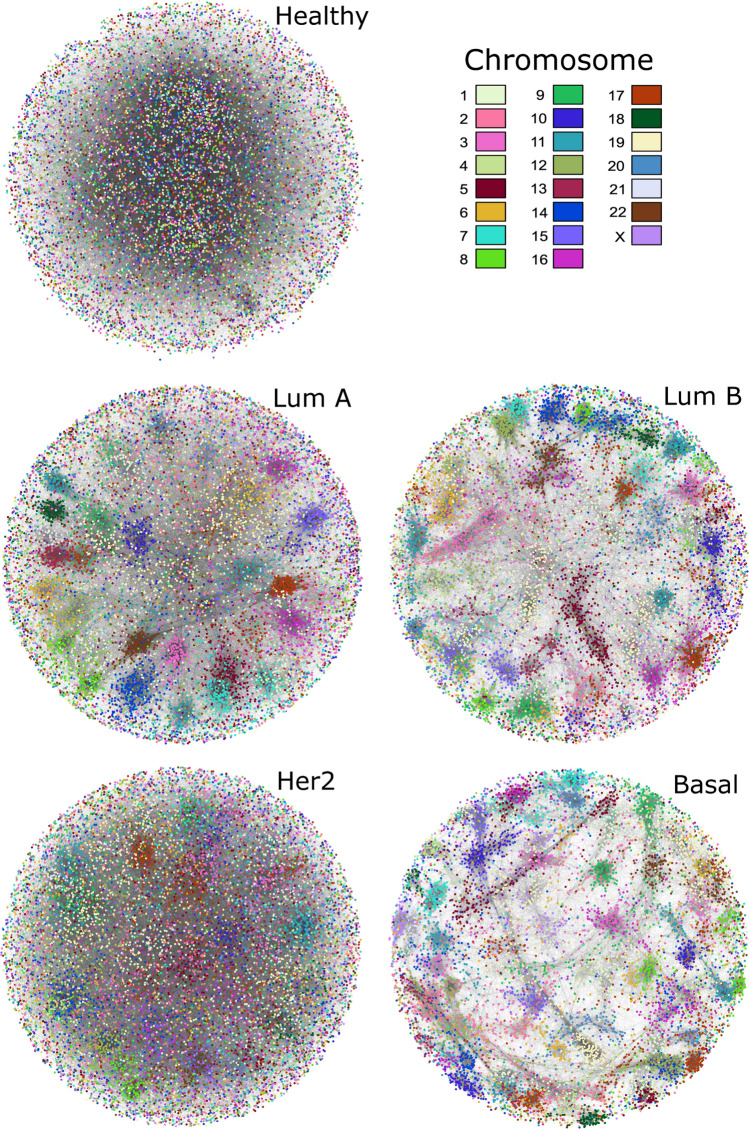
Network structure of the top-100,000 highest interactions. Starting from the full transcriptomes for the 4 breast cancer subtypes and the healthy breast tissue as measured by RNA Sequencing, mutual information calculations were used to infer the 5 associated networks. Once statistical significance was determined and thresholding was performed, networks of comparable size (with n = 100,000 interactions, ordered by statistical significance) were built. For this visualization, genes were colored according to the chromosome they belonged to. Network visualizations were performed using the spring embedded algorithm. In cancer networks, clusters of genes from the same chromosome (same color) are visible.

The top-100,000 highest MI interactions in the five phenotype networks are depicted in Fig. 1. Visualizations of these first layers were performed using the *spring-embedded force directed* algorithm. Genes are colored according to the chromosome each gene belongs. A trend of genes being placed close to other genes from the same chromosome is observed in the four breast cancer subtypes. It is also noticeable that healthy network shows edges more homogeneously distributed between chromosomes.

It is worth noticing that each subtype presents a different structure. For example, despite all networks contain the same number of interactions, Basal subtype GCN shows less vertices, since those edges are joining genes that form a cluster that is more connected among inside elements than outside genes. The following GCN in terms of *cis-*clusters is Luminal B, followed by Luminal A, and finally HER2+ network. Table 1 shows the *cis-* and *trans-*interactions for each GCN in the top-100,000 interactions.

**Table 1:** Number of *cis-* and *trans-*gene–gene interactions in the five GCNs.

Feature	Control	Breast cancer subtypes			
		Luminal A	Luminal B	HER2	Basal
*cis-*interactions	9186	89,274	99,721	77,081	102,607
*trans-*interactions	95,955	15,467	5020	27,660	2134
Total interactions	104,741	104,741	104,741	104,741	104,741

The remarkable differences between healthy and any subtype proportion in terms of *cis-* and *trans-*gene–gene interactions have been previously reported^16–20,56^. Once we observed the differences of intra and inter-chromosome interactions between cancer and control phenotypes, we wanted to analyze the relevance of *cis-* and *trans-*edges in terms of network connectivity.

**Table 2:** Structural features of the top100,000 GCNs* k-cores*.

Feature	Control	Breast cancer subtypes			
		Luminal A	Luminal B	HER2	Basal
Number of components	2	1	5	2	14
Number of modules	5	9	12	4	14
Number of inter-module links	3643	360	12	5	0
Number of genes	546	765	842	278	701
Total interactions	16,502	24,538	22,352	7017	14,746

### *trans-*interactions in cancer are relevant for network maintenance

**Figure 2:**
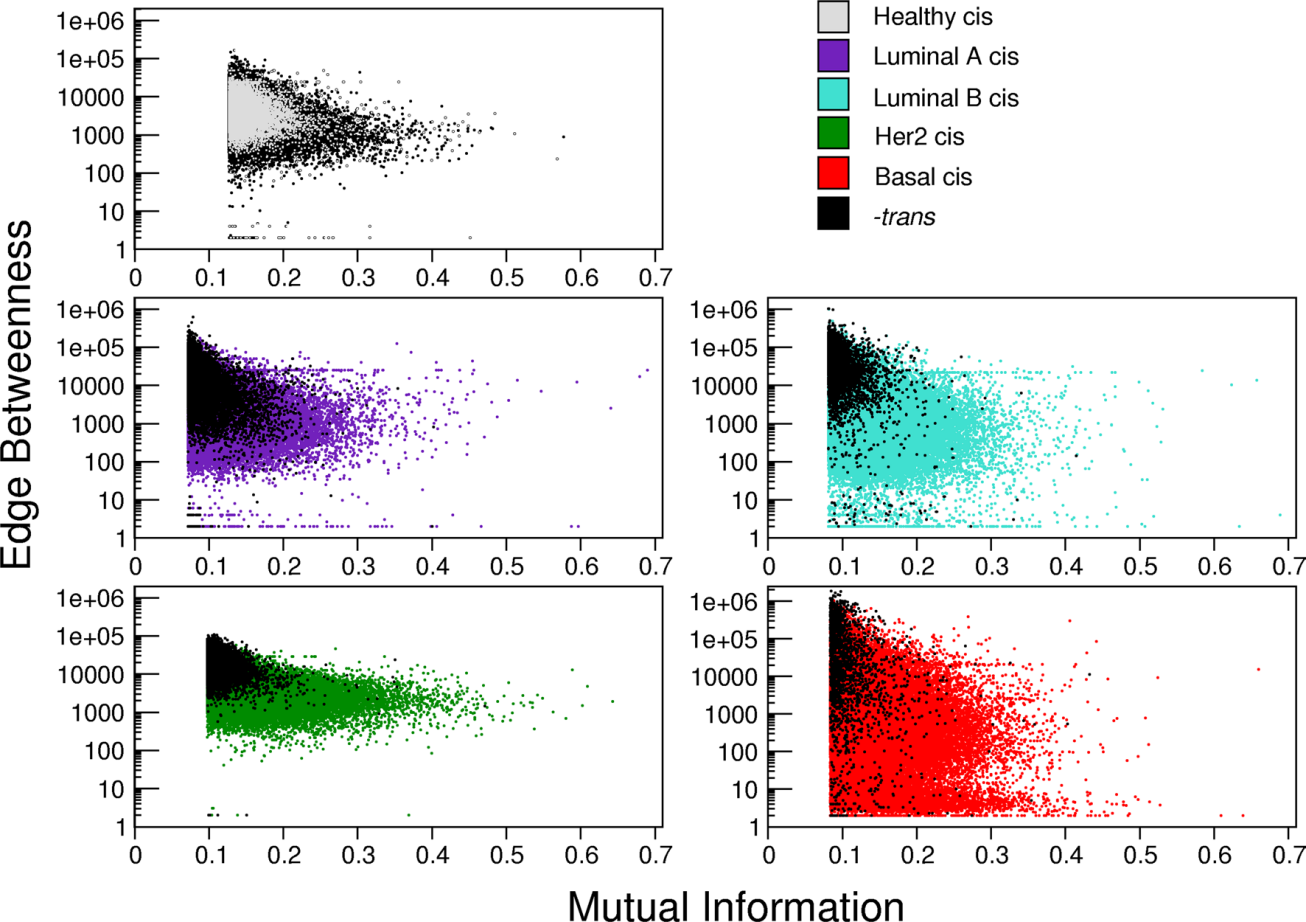
Scatterplots of co-expression mutual information (MI) values vs. Edge Betweenness (EB). In this representation, colored dots represent *cis-*interactions for each breast cancer subtype, as well as for healthy network. Black dots show *trans-*interactions for any phenotype. Notice that for cancer scatterplots black dots appear in the highest EB values (Y-axis), meanwhile colored points are somehow homogeneously distributed.

In Fig. 2 we show scatterplots representing the distribution of edge betweenness for all gene–gene interactions depending on their MI co-expression values for the five GCNs. Inter-chromosome (*trans-*)edges are depicted in black for all networks, meanwhile, each network has a different color to represent *cis-*interactions. It is noticeable that in healthy GCN black dots are more broadly distributed in the X-axis, showing that the edges with the highest co-expression values (MI) are formed by *trans-*interactions.

In the case of breast cancer subtypes, despite the *trans-*interactions in the GCNs do not have the largest MI values, the values of edge betweeness (EB, a network centrality measure defined as the number of the shortest paths that go through an edge) are higher than *cis-*ones. This result could be relevant in functional terms, because *trans-*interactions appear to maintain the already diminished communication in the cancer networks: *trans-*interactions maintain cohesion in all the five phenotype GCNs.

It is also worth noticing that the distributions of MI and EB values are not similar among phenotypes. For instance, in the basal subtype, the distribution of EB values is broader than in any other subtype. This could be due in part, to basal subtype GCN containing the largest amount of *cis-*interactions of the four breast cancer subtypes.

On the other hand, HER2+ subtype GCN has the most narrow distribution of EB values, independently of the type of interactions (*cis-* or *trans-*). Finally, the distribution of EB and MI values in the control GCN behave opposed again, compared with the cancer GCNs. *trans-*interactions surround the *cis-*ones, i.e., highest and lowest EB values came from genes in different chromosomes.

Once the top layers of the five phenotypes were analyzed, we extracted the 30-core of all GCNs to observe whether or not the *cis-/trans-*proportion was also maintained, and also whether a given *k-core* could provide more information regarding the spatial distribution of the co-expression patterns in health and disease networks. In what follows, we will present a spatial and chromosomal analysis of the 30-cores in the five phenotypes.

### 30-core of healthy network is composed of *trans-*interacting genes

**Figure 3:**
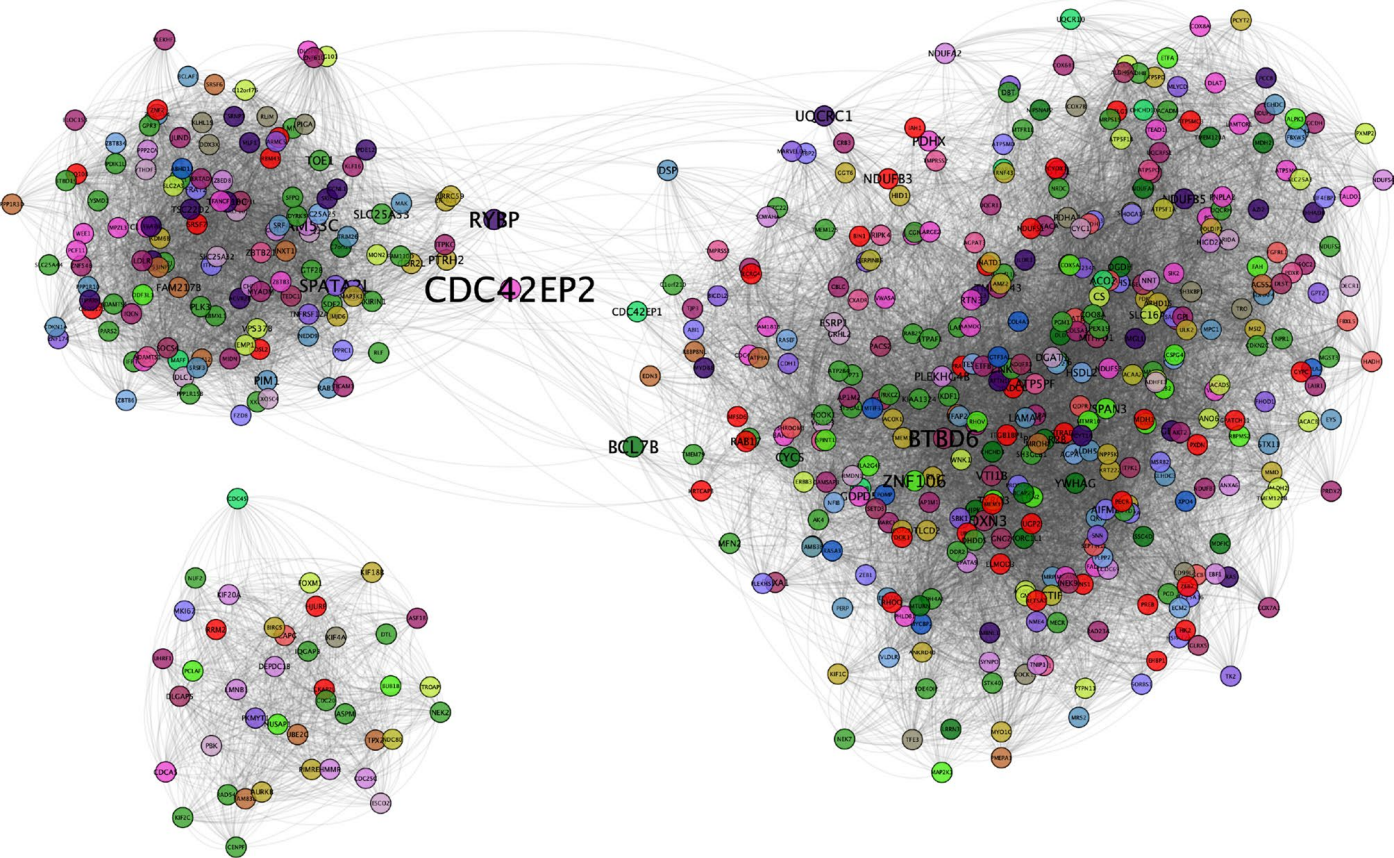
30-core healthy GCN. In this representation, genes are colored according to the chromosome they belong to.

The 30-core in healthy network, $Healthy_{30}$ (Fig. 3) is composed of 546 genes from all chromosomes (except for chromosome Y), and 16,502 interactions. This core has two components with the largest one formed by 505 nodes. The largest module is split into two communities.

This kind of modular behavior is also observed in all breast cancer subtypes, indeed in a more remarkable way. In the rest of cases, network 30-cores are separated into components with no further division, and also, said components are almost totally *cis-*, except for the case of the Luminal A network.

### The Luminal A network 30-core contains one *trans-*module

**Figure 4:**
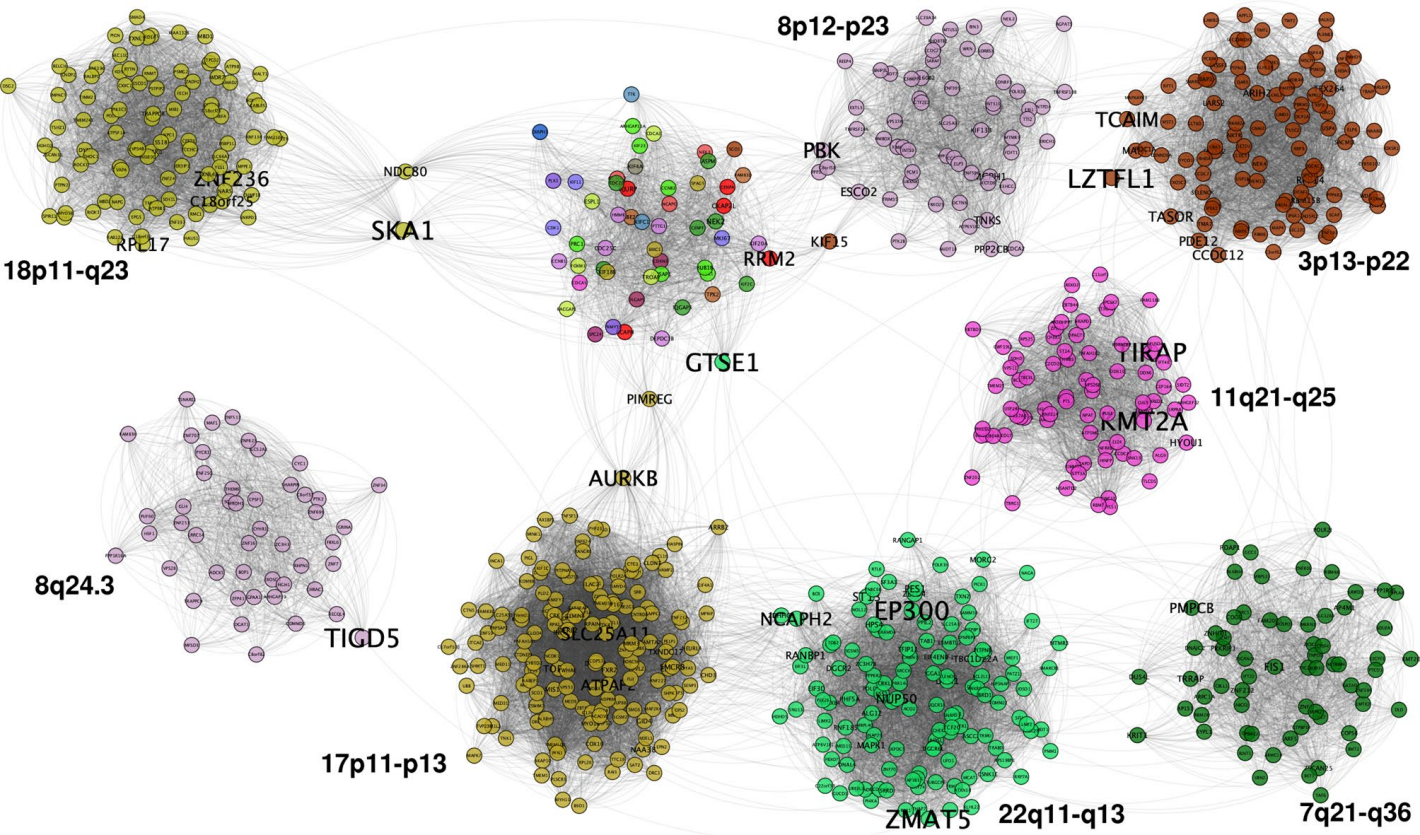
Luminal A 30-core network. In this case, a giant component is divided into nine clearly separated modules. The size of gene names is proportional to the betweenness centrality of genes. Aside of each module, the chromosome region in which those genes are located is depicted. To notice that genes with highest betweenness centrality are those that join communities, such as the case of SKA1, AURKB, TIGD5, or LZTFL1.

In the case of the Luminal A 30-core network $LumA_{30}$, this network has one giant component. However, this is divided into 9 separated communities (Fig. 4). Community structure of $LumA_{30}$ GCN is clearly divided into *cis-*gene clusters except for one, composed of genes from any chromosome. Apart from that module, the rest of them are located at chromosomes 11, 17, 18, 22, 3, 7, and 8. Additionally, it can be observed from the figure, that *cis-*modules are not only intra-chromosomal, but also they belong to neighboring cytobands. Regarding Chr8, two different components appear on it. However, they are topologically separated, i.e., those communities do not share interactions. One of the chr8 modules contains genes exclusively from the 8q24.3 region, meanwhile the other one is formed by genes from 8p12–8p23.3 region.

### Modules in the 30-cores of breast cancer GCNs have physically close genes

**Figure 5:**
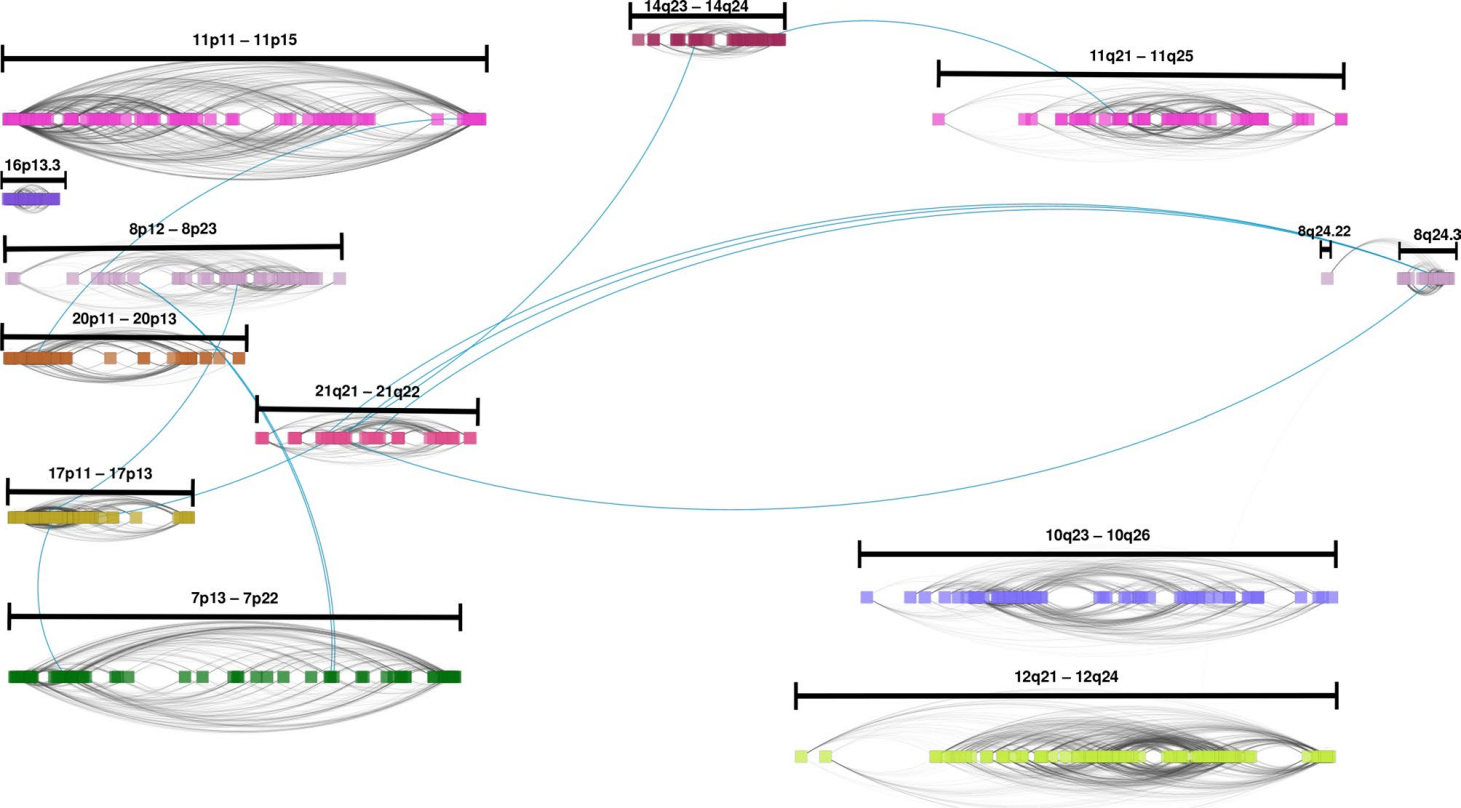
30-core of Luminal B breast cancer network. In this ideogram representation, genes are placed according to their starting base pair. Black straight lines cover the chromosome bands ocuppied by those clusters. It can be appreciated that all modules are *cis-*complexes; gray links represent intra-module links, meanwhile blue thicker lines show inter-module relationships. Separated clusters from the same chromosome, such as Chr11 and Chr8 also appear. Just as in the case of Luminal A chromosome 8 clusters, one of them contains genes from Chr8q24.3 region, while the other cluster is covered by genes from 8p12 to 8p23.3 region.

In the case of $LumB_{30}$ GCN, five connected components form the core, but in this case, modules are strongly separated (only 12 inter-modules links). Modules in this case are composed of *cis-*only interactions. Besides all modules have interactions within one-chromosome each, those genes are even physically close among them. The chromosome location of those genes lies in contiguous or even the same cytoband. Figure 5 show the cytobands in which genes from each module of $LumB_{30}$ GCN are placed.

In the case of Chr8 and Chr11, two separated clusters appear. In both cases, one cluster belongs to the p-arm and the other one is formed by genes in the q-arm of their respective chromosome. Additionally, it is worth noticing that these clusters in the same chromosome do not have joining edges.

### Differential gene expression do not induce modular structure in HER2+ network

Taking into account that (a) *k-cores* may represent those nodes that function as a backbone of the whole network^13^, and (b) the 30-core networks of the breast cancer subtypes are formed by physically close genes, investigating the differential gene expression of each cancer phenotype respecting the control one resulted appealing.

**Figure 6:**
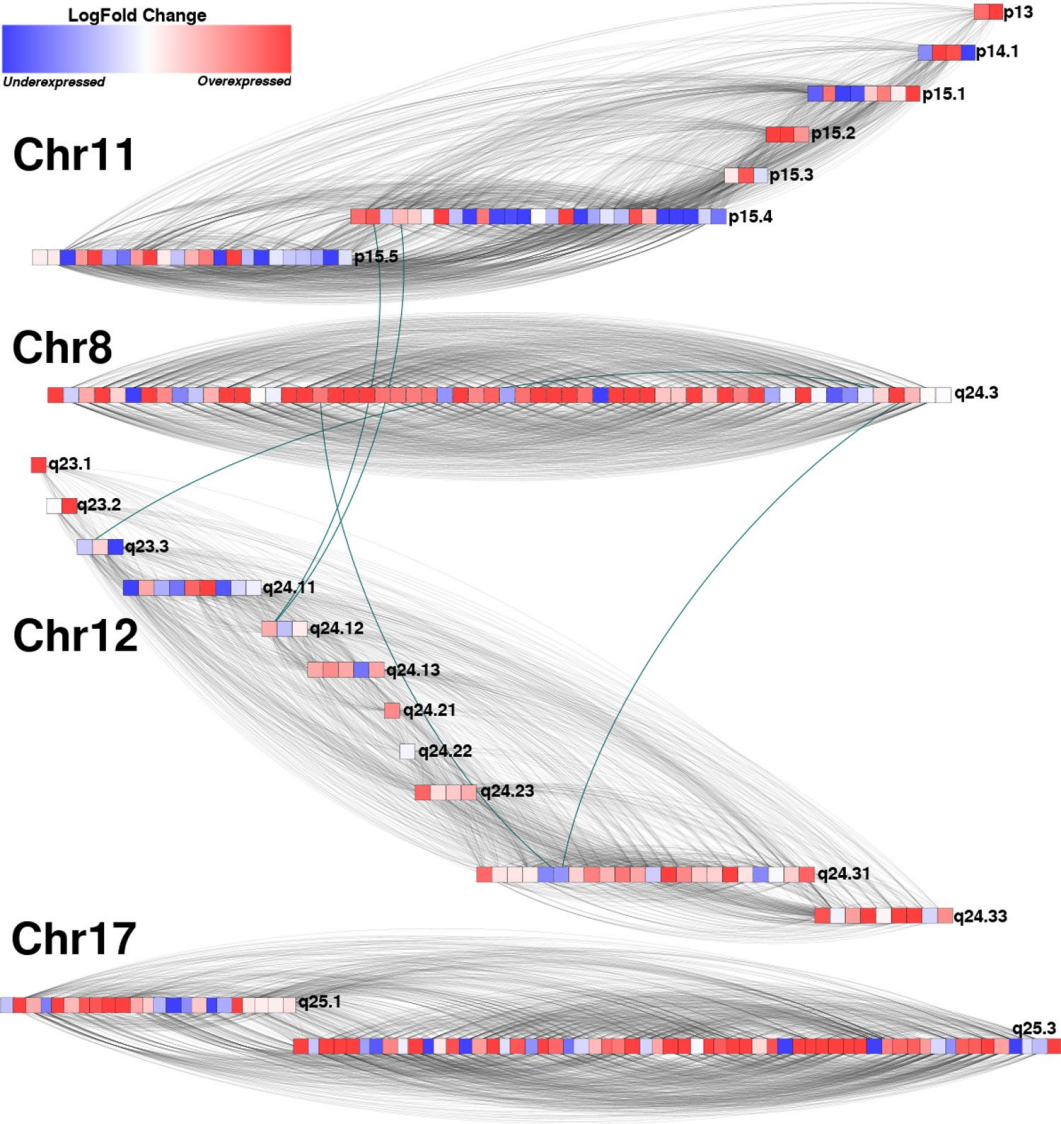
HER2+ 30-core network. In this representation, genes are colored according to their differential expression. Red squares represent overexpressed genes, and blue, underexpressed ones. Grey links join *cis-*interactions, and blue thicker lines join genes from different chromosomes (*trans-*interactions). Each row of genes contains elements from the same cytoband. Each chromosome in the figure forms one community: Chr11, Chr8, Chr12 and Chr17.

An important question is whether the spatial distribution of 30-core genes follows a trend of similar differential expression pattern, i.e., those neighbor genes inside a community are biased to be mostly overexpressed or preferentially underexpressed. This may point out to *regional* mechanisms of transcriptional control such as gene expression *bubbles* or the action of the same set of transcription factors.

Figure 6 shows the case of $HER2_{30}$ network. There, the network has two separated components, One formed by Chr17 genes exclusively, and the other one composed of genes from Chr8, Chr 11, and Chr12. As in Luminal B 30-core network, communities in the 3-chromosome component, are clearly separated in physically close genes.

In Fig. 6, gray edges show intra-community interactions. Interestingly, these links are also *cis-*relationships. In the figure, genes in one row represent those belonging to the same cytoband. Inside a row, genes are sorted by the gene start bp. Color of genes depends on its differential expression. Overexpressed genes are colored red, meanwhile blue squares represent those with underexpression.

One could expect that according to the physical closeness between intra-community genes, those genes would have a similar differential expression pattern. However, that is not the case of $HER2_{30}$ network. The more coarse-grained separated components, show genes with both differential expression trends. Communities are not separated into overexpressed or underexpressed sections. Finally, as in $LumB_{30}$ case, genes are connected with neighboring genes, from the same or close cytobands.

### Basal 30-core network interactions are mostly intra-cytoband

**Figure 7:**
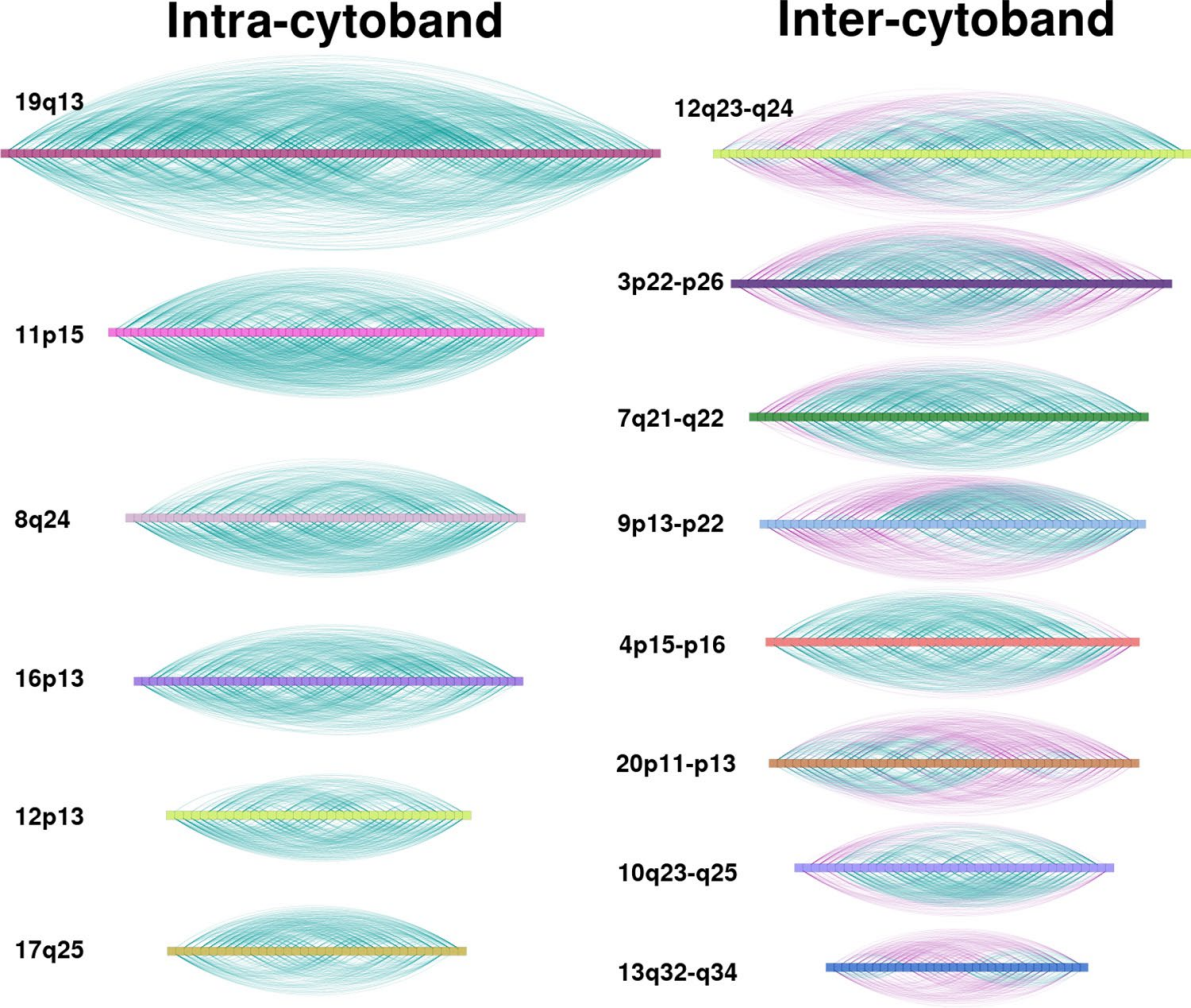
Basal 30-core network. In this representation, the 14 separated components obtained from the 30-core network are depicted separately. Genes are colored according to the chromosome to which each gene belongs. Cyan interactions join intra-cytoband genes, while inter-cytoband edges are depicted in purple.

$Basal_{30}$ is composed of 14 *cis-*components, which are located in 13 different chromosomes (Fig. 7). There are two clusters from chromosome 12. One from region p13, and another one from q23–q24 region. Interstingly, the 30-core network from Basal subtype has not even one inter-chromosome interaction. All the 14,746 edges are *cis-*.

Additionally, all components are formed by genes from one chromosome, but more importantly, from the same cytoband, or neighboring cytobands. Figure 7 depicts the 14 components of Basal 30-core network. In the figure, intra-cytoband links are depicted in cyan, meanwhile purple lines join inter-cytoband (but *cis-*) gene–gene relationships.

It is noticeable that the majority of edges are intra-cytoband, even in those components with genes from different cytobands. This is the 30-core network with more separated components (14), but also the one with more intra-cytoband interactions inside its components.

### *cis-*communities are not associated with any biological process

After detecting all communities in the *k-core* networks (Supplementary Fig. S2), we evaluated their functional role, by means of an over-representation analysis for each inferred community. Importantly, as it can be observed in Table 2, and in Supplementary Fig. S2, the number of modules for each *k-core* network is reduced, and in all cases for cancer networks, such modules are composed of *cis-*interactions, except for one community in $LumA_{30}$ GCN.

**Figure 8:**
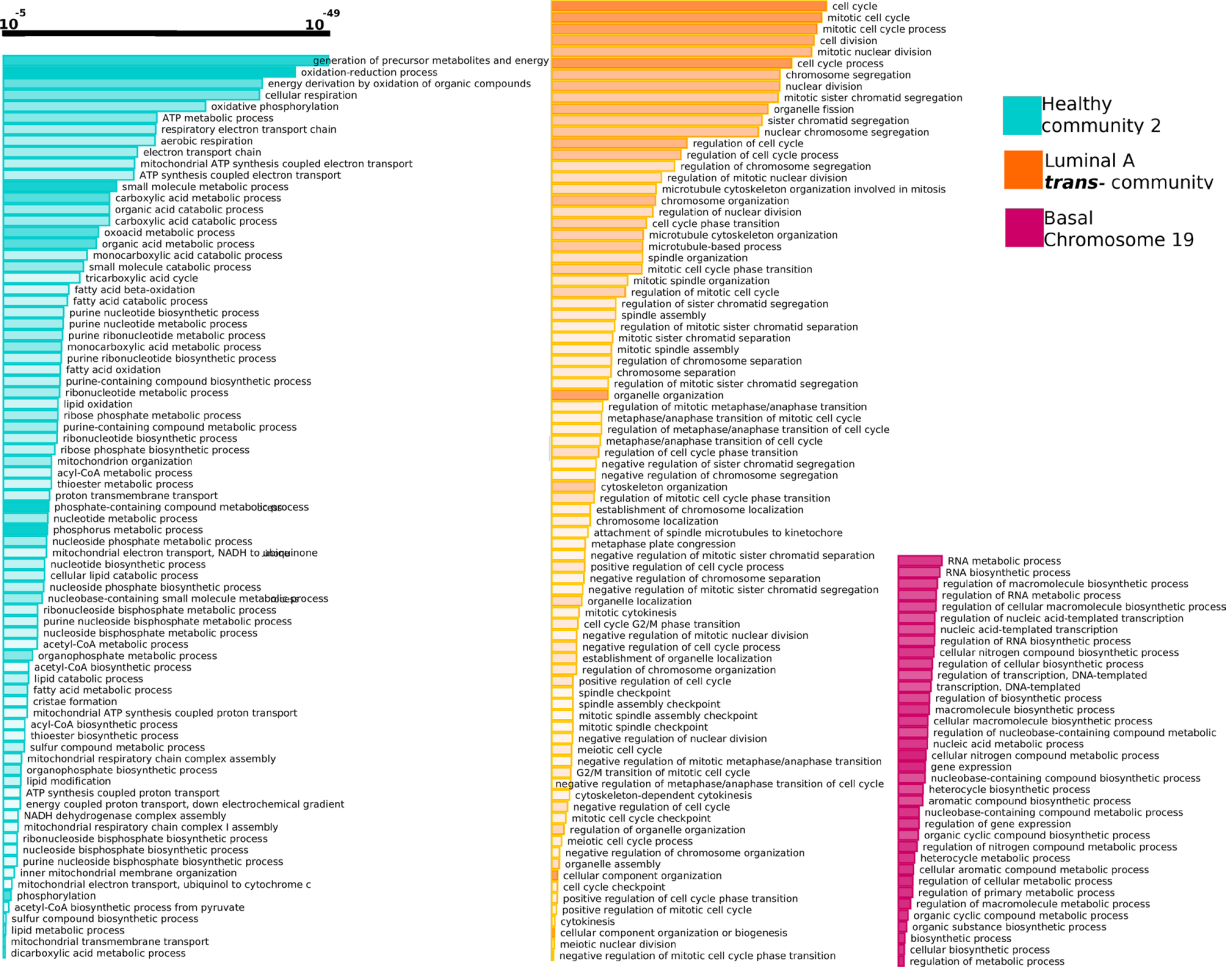
Functions associated with specific *k-core* communities. Here we can appreciate all enriched processes obtained from the over-representation analysis for the five *k*-core networks. Despite 44 communities are found in these networks, only three of them have associated processes. Length of bars is proportional to the significance of such process. Opacity of bars is proportional to the number of genes in the community that intersect with the associated process.

To note, the total amount of communities in the five *k-core* networks is 44. However, only three communities are associated to a particular set of biological functions: one for the $Healthy_{30}$, the *trans-*community of $LumA_{30}$, and the Chr19 module from $Basal_{30}$. In Fig. 8 the enriched processes of the three communities are depicted. There, it can be observed the following:

- The three communities have different associated processes. None of the depicted biological functions are shared between communities.
- Healthy and Luminal A-associated processes are highly enriched (*p*-value$<10^{-40}$).
- Functions associated with metabolism appear in healthy network.
- Cell cycle-related processes are present in Luminal A network.
- Regulation of gene expression processes appear in Basal Chr19 network.

**Figure 9:**
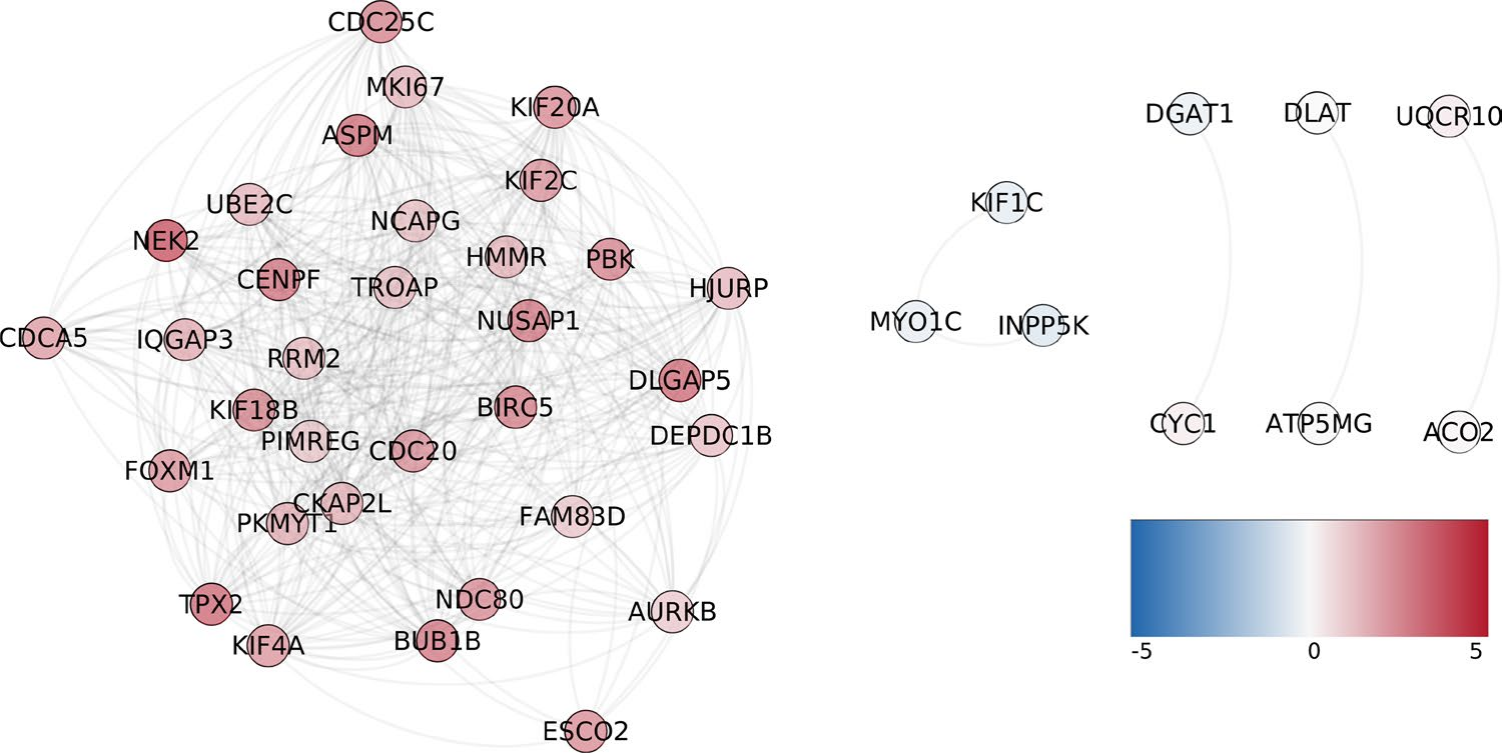
Intersection between healthy and $LumA_{30}$ networks. This network shows the shared interactions between the aforementioned networks. Genes are colored according to its differential expression LogFC value: red genes are overexpressed, while blue genes have negative LogFC values. Importantly, this cluster is composed of only *trans-*interactions.

Interestingly, among all modules observed in the 30-core networks, there is a subset of those that are shared between different phenotypes. The 30-core subnetwork of healthy phenotype does not share genes or interactions with any other phenotype’s network but the Luminal A (Fig. 9). The shared genes and links are an important part of the *trans-*subset in Luminal A. Furthermore, all genes in the main component in this intersected network are clearly overexpressed. Genes such as MKI67, CDC25C, KIF20A and many other oncogenes are present in this intersection. Additionally, this subset is associated with cell cycle and cell division processes (Supplementary Table S1).

### There are shared *cis-*clusters between cancer phenotypes

In the case of cancer 30-core intersections, the shared interactions are part of *cis-*clusters. $LumA_{30}$ shares only *cis-*interactions (5925) with Luminal B network in gene-pairs from chromosomes 8, 11 and 17. Specifically, two clusters from Chr8, one of them from Chr8q24.3, and the other from Chr8p12–21.3; the Chr11 cluster from Chr11q22.2–q25, and finally Chr17p11–p13. $LumA_{30}$ also shares 923 links with $HER2_{30}$. All interactions are located at Chr8q24.3. With $Basal_{30}$, $LumA_{30}$ shares 769 links from Chr8q24.3 region, and 552 from Chr 7q22.1–22.3. Fraction of *cis-*interactions remained significantly smaller for Luminal A when using other *k* values for the computation of the *k*-core, including $k=10, 15, 20, 25$ and 35 (Supplementary Fig. S3).

**Figure 10:**
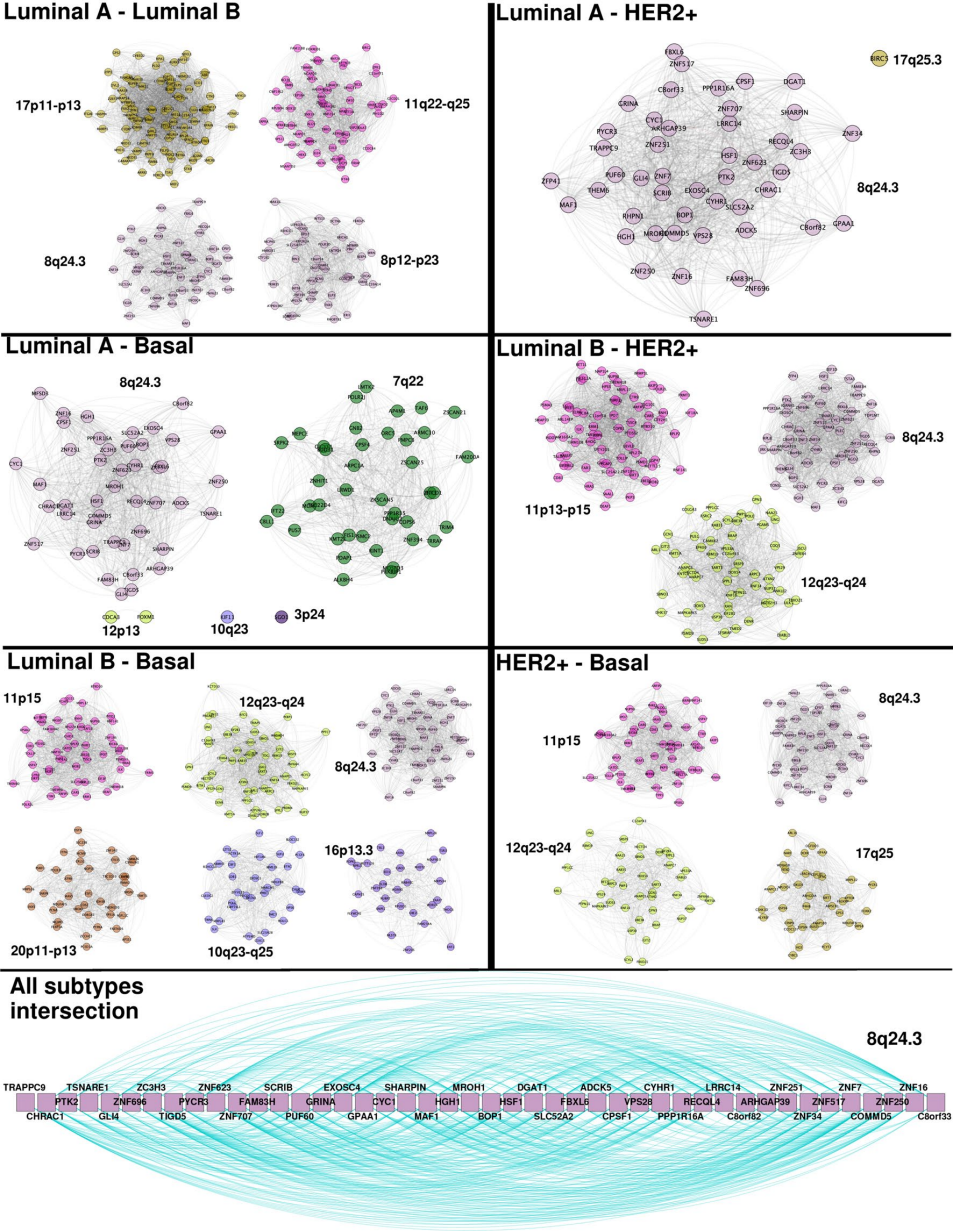
Breast cancer 30-core networks intersections. In each image, edge intersection for two different breast cancer subtypes is depicted. The bottom image shows the intersection of the four phenotypes. It is important to remark that intersection is at the interactions level. The only set of adges that is shared among the four 30-core network of breast cancer subtypes is composed of genes in the Chr8q24.3 region.

For $LumB_{30}$, aside from the 5925 links shared with $LumA_{30}$, 4055 are common between this phenotype and $HER2_{30}$. The clusters between them are from Chr11p13–p15, Chr12q23–q24, and Chr8q24.3. In the case of its intersection with $Basal_{30}$, 4720 links between them exist. Unlike the other intersections, in this case $LumB_{30}$ and $Basal_{30}$ share links from six different chromosome locations: Chr11p15, Chr12q23–q24, Chr8q24.3, Chr20p11–p13, Chr10q23–25, and Chr16p13.3. $HER2_{30}$ also shares 3310 interactions with $Basal_{30}$, these are from Chr11p15.2–p15.5, Chr 17q25, Chr8q24.3, and Chr12q23–q24. All these intersections are depicted in Fig. 10.

As it can be appreciated from the paragraph above, the only set of genes that is shared among the four breast cancer subtype 30-core networks is the one composed of genes in Chr8q24.3 (Fig. 10). We also performed an over-representation analysis of this gene set. It is worth mentioning that these shared genes are not associated with any GO category.

### Core genes remain across co-expression layers in healthy, but not in cancer

The only breast cancer network whose 30-core share genes from the 30-core of healthy tissue is $LumA_{30}$. As shown, this group of genes form a connected component conformed of genes in all chromosomes, and they are overexpressed. This contrasts with the connected components found in the other 3 cancer tissues, only containing genes in one or two different chromosomes each. Previously, it has been reported that genes in the main core of healthy tissue are re-configured in sub-layers of co-expression, i.e., across layers of 100,000 co-expression interactions^14^. Importantly, this re-configuration is only obtained in healthy tissue and not in cancer, thus suggesting that genes in the main core of the network are essential to the proper functioning of the cell. Here, we investigate whether shared genes between $Healthy_{30}$ and $LumA_{30}$ are conserved across co-expression layers in both tissues. A high conservation rate in healthy tissue may imply a reminiscent of essential genes in Luminal A lost in all other tissues.

**Figure 11:**
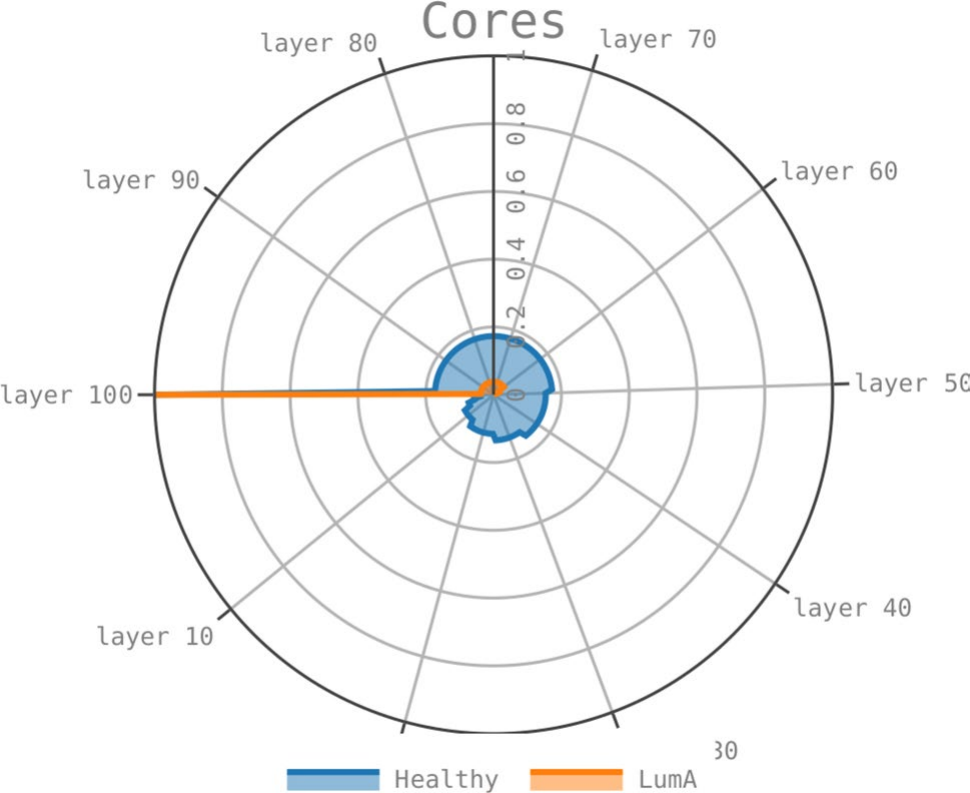
Conservation rate (*ccr*) of genes in the intersection of the 30-core of healthy (blue) and Luminal A (orange) tissues across 100 co-expression layers of 100,000 interactions each. Layers of coexpression move in clockwise sense from layer 100 (left) to layer 0. *ccr* is indicated in the concentric circles, the outer circle represents a $ccr=1$, meanwhile a $ccr=0$ is placed in the center of the circle. Notice that for the case of LumA intersection, conserved genes in the following co-expression layers rapidly dissapear.

Figure 11 shows the conservation rate of common 30-core genes in Healthy and Luminal A tissues across 100 co-expression layers. Each layer consists of 100,000 co-expression interactions, layer 100 is the layer used throughout this work and consists of the top 100,000 co-expression interactions, layer 99 consists of the subsequent 100,000 co-expression interactions and so forth for layers 98 through layer 1.

In total, we consider 10 million co-expression interactions of healthy and Luminal A tissues. We observe a positive conservation rate across the first 40 layers in both tissues of their shared genes in the 30-core of the 100 layer. This conservation rate is around 4 times larger for healthy interactions compared to interactions in Luminal A.

In Luminal A, after layer 56, the conservation ratio drops to 0, meaning that a small part of core genes are conserved in the top 44 co-expression layers. On the contrary, in healthy tissue, genes are conserved across all 100 layers and show conservation rates greater than 15% for the first 50 layers. This suggests that genes common to both 30-cores are present due to healthy co-expression interactions conserved in Luminal A tissue but lost in the other 3 cancer subtypes.

The other group of genes we considered to study across layers is the intersection set of the four 30-cores of cancer-subtypes. This set consists of 44 genes in chromosome 8. The conservation rate of these genes in all 4 sub-types is 0 after the top layer. Starting in the second layer, these genes are not found in the subsequent cores in the co-expression layers of any cancer sub-type.

### *cis-*cores prevail at different values of *k*

In order to evaluate whether our results were not biased due to the selected value of *k*, we looked at the *cis-*/*trans-*distribution of interactions across six different core numbers, namely *k* equal to 10, 15, 20, 25, 30, and 35. We found that core *cis-*edges (genes interactions within the same chromosome) are weak for all types of cancer. The core of Luminal A has a consistent percentage of inter edges, with a mean of 7.3% and standard deviation 0.8. The cores of other subtypes lose almost completely all *trans-*edges after core number 10, suggesting that results shown for core number 30 are conserved through different core numbers (Supplementary Fig. S3).

## Discussion

The five top 100,000 GCNs are different. This has been previously observed in breast cancer subtypes by using different technology such as microarrays^10,11,57^. Recently, by using TCGA-derived RNA-Seq data, we shown that the number of *cis-/trans-*rate (the ratio between number of *cis-*edges over *trans-*ones), decreased in the following order: $healthy \rightarrow LumA \rightarrow LumB\rightarrow HER2+\rightarrow Basal$^19^. This rank coincides with prognosis and, in some cases, aggressivity of the subtype. However, it can be observed from Fig. 1 and Table 1, the first top-100,000 breast cancer networks show a different order in the *cis-/trans-*rate: $healthy \rightarrow HER2 \rightarrow LumA\rightarrow LumB \rightarrow Basal$.

Notwithstanding this, in the case of the 30-cores, the trend returns to the initial order, as it can be observed in Table 3. This may be because at the highest MI interactions, not the top-100,000, but even higher, this structure reflect the actual determinants of the co-expression landscape. Then, the nature of those interacting elements in the 30-core is a reflection of the most important interactions for each phenotype. And the fact that Luminal B, HER2+ and Basal 30 cores have less than 20 *trans-*edges, may suggest how the actual backbone of the top-100,000 interactions GCNs in those phenotypes is completely deprived of long-range interactions.

**Table 3:** Number of *cis-* and *trans-*gene– gene interactions in the five 30-cores of top-layer GCNs.

Feature	Control	Breast cancer subtypes			
		Luminal A	Luminal B	HER2	Basal
*cis-*iInteractions	1071	23,371	22,340	7015	14,746
*trans-*interactions	15,431	1567	12	5	0
*trans-*percentage	93.5%	6.16%	0.053%	0.071%	0%
Total interactions	16,502	24,938	22,352	7017	14,746

On the other hand, in $LumA_{30}$ (often considered the less aggressive subtype of breast cancer), the appearance of one cluster with *trans-*interactions may show some remaining functionality similar to a healthy phenotype. However, the rest of the 30-core is composed of *cis-*clusters. Importantly, this set of genes is remarkably overexpressed, and they are significantly associated with mitotic cell division; perhaps indicating the upregulation of these functions.

The fact that highest edge betweenness values in cancer networks correspond to *trans-*interactions, reflect that those inter-chromosome links are joining modules, and these modules contain in its majority *cis-*interactions. In some sense, *trans-*edges are more relevant to maintain the network global structure, at least in the first layer of co-expression.

Now, regarding spatial distribution of genes in the 30-core of breast cancer subtype GCNs, several features can be observed that call for attention. For instance, in the case of $LUMA_{30}$, 8 out of the 9 clusters are *cis-*, and from them, 7 out of 8 are composed of genes in the same chromosome arm. The only case in which a cluster is formed by genes from both arms is Chr18 cluster (Fig. 4).

The latter could be indicative of two phenomena: (i) genes interact more frequently in the same chromosome arm, maybe due to the fact that the centromere constitutes a physical barrier to prevent gene co-expression, and (ii) Luminal A subtype conserves long-range interactions, between arms of the same chromosome, and between genes from different chromosomes. However, even in Luminal A subtype the majority of clusters are composed of physically close genes. This coincides with a recently published work from our own group^19^. There, we stated that gene *cis-*co-expression decays with distance in breast cancer subtypes. In this work, we reinforces that hypothesis showing that intra-arm interactions are much more frequently in breast cancer 30-cores.

The remaining *trans-*cluster may indicate a functional interaction between those genes, and the associated function to that cluster could be relevant for cell maintenance. The same could occur between inter-arm interactions in Chr18. It is worth to mention that Luminal A is the only subtype that contains genes from Chr18 in its 30-core. A comprehensive analysis in those terms is necessary to assess the role of those interactions in a functional context.

The finding of inter-chromosomal clusters in a breast cancer network derived from the Luminal A subtype has been previously observed in smaller networks (13,000 edges)^19^ There, it was demonstrated that the only breast cancer subtype with a network component in which trans-interactions are predominant, is Luminal A. In a different approach^58^, we also observed how a *trans-*component in the Luminal A network contains communities which are associated with biological processes. The appearance of one cluster with *trans-*interactions may show some remaining functionality similar to a healthy phenotype.

We observed a higher proportion of intra-cytoband interactions in breast cancer in the top-100,000 breast cancer networks. The separation between genes from different cytobands leaded us to argue whether those separations could be related to their differential gene expression. This is not the case, at least in the 30-core networks.

The fact that differential gene expression is not a factor that separate clusters is also matter of further research. The correlation measure used here is Mutual Information, which shows the statistical dependency between expression of any two genes. One may suppose that clusters of genes could be highly correlated because those genes present similar expression signatures. This is not the case, except for a reduced group of clusters, in which we can found the only *trans-*cluster in breast cancer, observed in Luminal A subtype. This reinforces the hypothesis that the *trans-*cluster is relevant for functionality in Luminal A breast cancer subtype.

Regarding the amount of intra-cytoband interactions in *cis-*clusters (Fig. 7), 6 clusters are totally intra-cytoband and the other 8 have a combination of intra and inter-cytoband edges. Interestingly, except for Chr13q cluster, the rest of clusters contain more intra-cytoband interactions. This reinforces the hypothesis in which gene co-expression is distance-dependent in breast cancer^19^.

In the case of edge network intersections between breast cancer subtype 30-core networks, it is remarkable that the only subset of shared genes and relationships is the one composed of Chr8q24.3 genes. It has been widely reported that region 8q24 is highly amplified in several types of cancer^59–62^.

Despite that most information regarding amplification of Chr8q24 and its association with cancer has been documented with respect to genes such as MYC or NOV^63–65^, in this work we observed a common feature in the four subtypes of breast cancer associated to the extreme region of the chromosome: q24.3. With regard to this particular region, PTK2 gene has been reported to be amplified in head and neck oral sarcoma^66^. PTK2 gene appears in the shared genes of 8q24.3 network (bottom of Fig. 10, third gene from the left).

Network structure analyses also reveal that in cancer, *cis-*interactions are more abundant in the first layers, but decay rapidly in the following layers. This can lead us to hypothesize that for the tumor phenotypes, the *trans-*links in the first layers collaborate to maintain cohesion in the network.

Even if the apparently random connectivity patterns in top layers of the tumor networks may suggest a lack of structural organization, a closer look reveals that organizational principles persist at certain levels: The four 30-core networks in breast cancer intersect in the region Chr8q24.3. Perhaps local co-expression is indeed a conserved phenomenon in cancer, and there may be a (yet unknown) underlying physical mechanism generating those local co-expression patterns.

This latter may be evident since despite the high molecular heterogeneity in cancer, there is a highly interconnected subset of genes that belong to a quite small region in a short chromosome, in all breast cancer molecular subtype *k-core* networks. The fact that this phenomenon is present all breast cancer subtypes considered raise a question as to whether this may be a more general cancer feature, even perhaps a hallmark of cancer.

Regarding the functional features found in the detected communities, we want to stress three major points: (i) Only three out of 44 communities were enriched. (ii) From all the detected communities, only one *cis-*community resulted functionally enriched (basal, chromosome 19). (iii) The other two communities are inter-chromosome and have the most significant values of enrichment.

The nature of the functions observed in the healthy network is mainly associated with metabolism and homeostasis. On the other hand, the case of luminal A *trans-*community is highly enriched in categories such as cell cycle, cell division and mitosis (see Fig. 8). Additionally, several genes located in that community were differentially overexpressed, supporting the hypothesis that for Luminal A breast cancer, the inter-chromosome interactions may favor the cell division process.

Cell-division-associated enriched processes in breast cancer has been observed elsewhere^67–70^. Recently, we found that in Luminal A breast cancer, the large majority of enriched terms are associated with nuclear division, DNA replication, chromatid segregation, and cell cycle checkpoints, i.e., cell division processes^58^. There, NUSAP1 gene was among the most relevant in terms of connectivity. Additionally, NUSAP1 has already been identified as a hub gene in a network of ER positive breast cancer tumor^71^. In this work, NUSAP1 is part of the trans-community, a cluster of 60 genes and it is connected to 56.

## Conclusions

A non-exhaustive list of results achieved with the network approach regarding breast cancer molecular subtype network structure is shown here:

- Top interactions of breast cancer networks tend to be intra-chromosome (*cis-*).
- *trans-*connections in cancer are important to maintain cohesion in the networks.
- Healthy network edges are mostly inter-chromosome.
- The *k-cores* of breast cancer networks are composed of neighbor same-chromosome genes.
- Luminal A *k-core* network has a *trans-*cluster, which is shared with the healthy *k-core*; genes of this cluster are overexpressed and participate in cell cycle-related processes.
- There are shared clusters between breast cancer k-cores. Those shared clusters are *cis-*.
- There is only one cluster shared between all cancer *k-cores*. This is located at Chr8q24.3 region.
- *k-core* genes are conserved across lower co-expression layers in healthy network, but in cancer there is no conservation of genes.

By using a multilayer network approach to GCNs, we have considered and analyzed the subnetwork formed by genes in the so-called 30-core. We were able to look at a form of structural blueprint behind the whole gene co-expression program in five phenotypes: one healthy network and four tumor networks corresponding to the different breast cancer molecular subtypes. The regulatory program is shown to be altered and gradually lost in breast cancer. This approach may be unveiling a mechanism for which the transcriptional program in cancer is completely altered during cancer manifestations. It is desirable though to gather further evidence of this phenomenon, possibly in other types of cancer, involving the use of other complementary omic technologies, in order to provide a more integrated, holistic and comprehensive gene regulation landscape of cancer. This may allow us to present more evidences about plausible mechanistic processes which may be affecting the global regulation of cancer cells.

## Supplementary Information

Supplementary Information.

Supplementary Figures.

Supplementary Table S1.

## Data Availability

The datasets analysed during the current study are available in the GDC Legacy Archive repository, https://portal.gdc.cancer.gov/legacy-archive. Programming code for this study can be found at the following GitHub repositories: https://github.com/CSB-IG/pychromnet, and https://github.com/CSB-IG/regulaciontrans-pipeline.
