# Supplementary material for: k-core genes underpin structural features of breast cancer
Source: Sci Rep. 2021 Aug 11;11:16284. doi: 10.1038/s41598-021-95313-y (PMC8358063; doi:10.1038/s41598-021-95313-y)
Supplement: Supplementary file 2 — Supplementary Figures. [file 41598_2021_95313_MOESM2_ESM.pdf]

k-core genes underpin structural features of breast cancer

Rodrigo Dorantes-Gilardi, Diana García-Cortés, Enrique Hernández-Lemus, Jesús Espinal-Enríquez

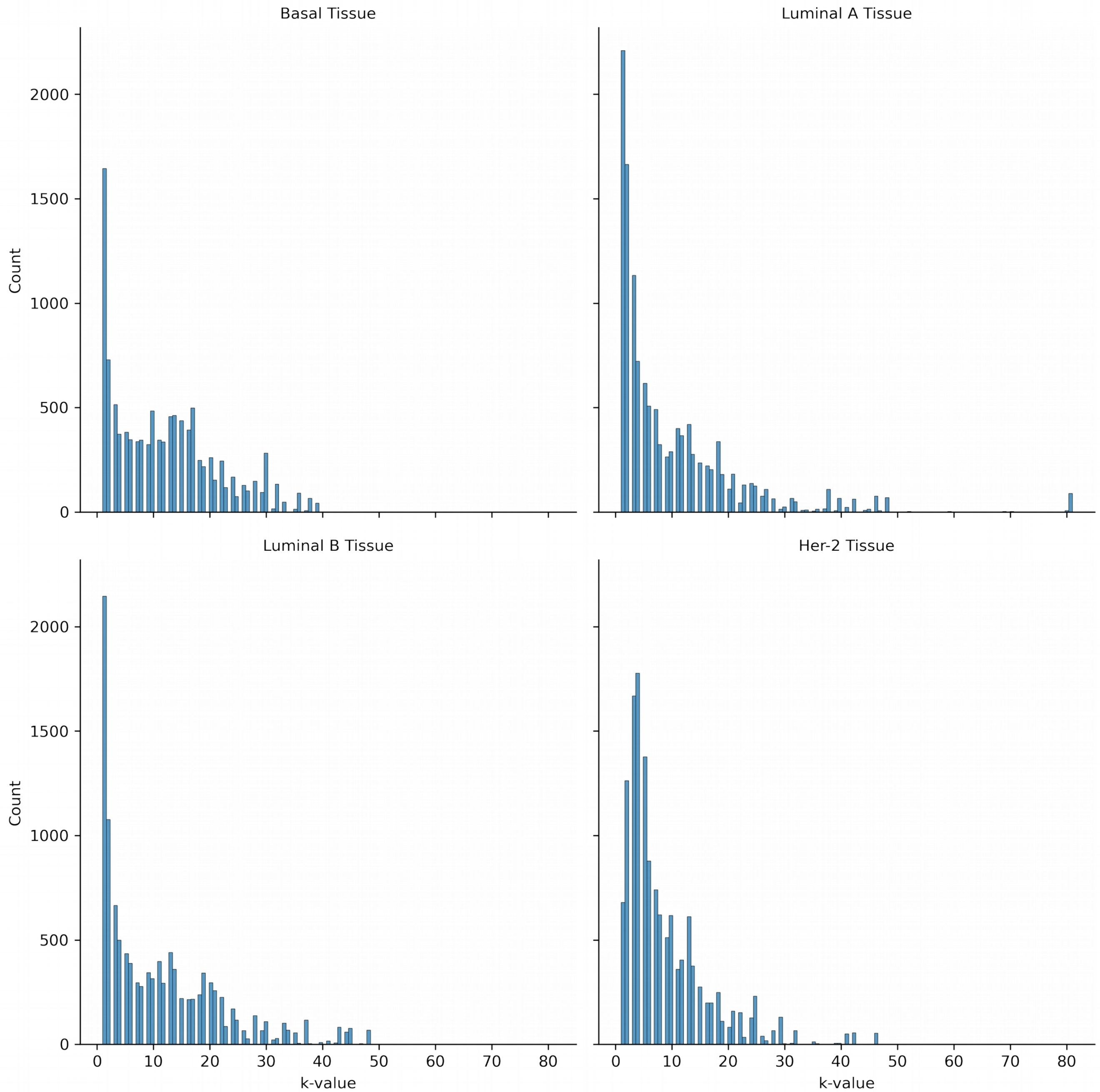

Supplemnetary Figure S1. Core number (*k*-value) distribution of genes across breast cancer subtypes. The core number of a node *v* corresponds to the greater value of *k* such that *v* is in the *k*-core of the network.

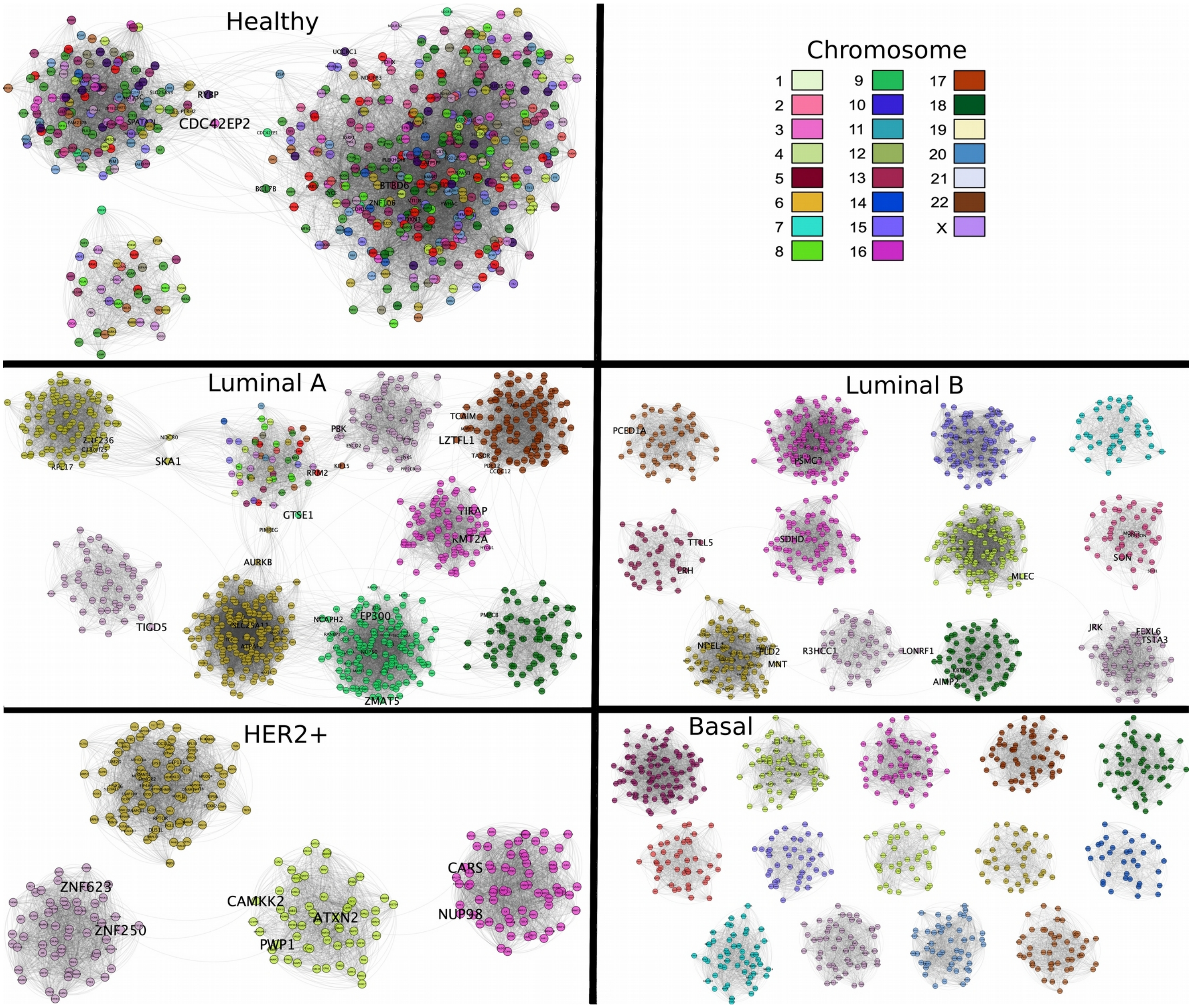

Supplementary Figure S2. Community structure of the five *k*-core networks. In this figure, nodes are colored according to the chromosome they belong. Each cluster of genes correspond to a detected community.

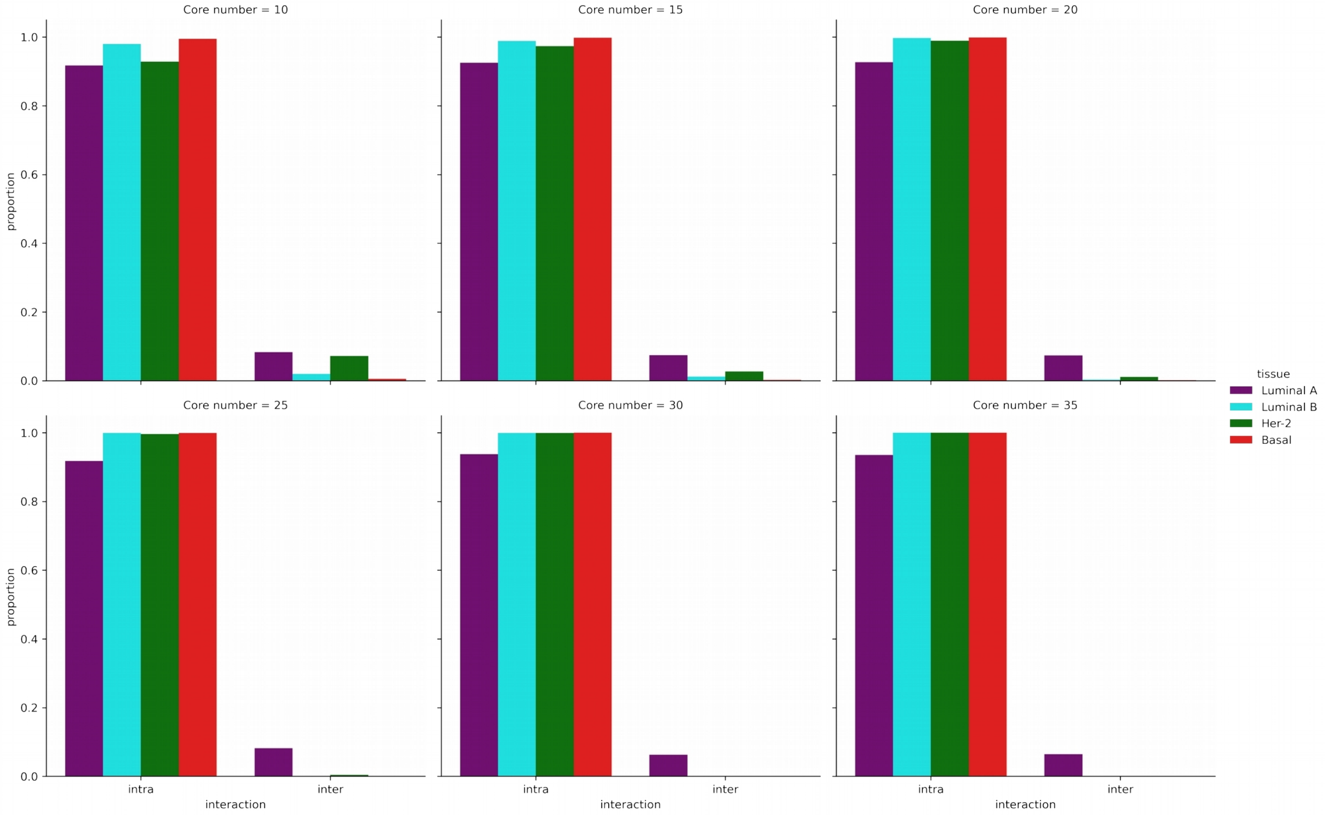

Supplementary Figure S3. *cis*- and *trans*- interactions for different values of *k* in breast cancer subtype networks. From top-left to bottom-right it is depicted the proportion of *cis*-interactions. The colors represent the different breast cancer subtypes. It can be noticed that for all cases, the*cis*- proportion is larger than the *trans*- interactions. Additionally, it can be observed that in all cases, the Luminal A core (purple bars) has less *intra-chromosome* edges, and consequently, more *trans*- links. The results presented in this work were taken from the 30-core case (bottom-center).
